# Presence of p24-Antigen Associated to Erythrocyte in HIV-Positive Individuals Even in Patients with Undetectable Plasma Viral Load

**DOI:** 10.1371/journal.pone.0014544

**Published:** 2011-01-18

**Authors:** Maria Noé Garcia, Maria Sol dos Ramos Farias, Maria Mercedes Ávila, Roberto Daniel Rabinovich

**Affiliations:** Microbiology Department, School of Medicine, National Reference Center for AIDS, University of Buenos Aires, Buenos Aires, Argentina; Institute of Infectious Diseases and Molecular Medicine, South Africa

## Abstract

**Background:**

HIV adherence to erythrocytes has been demonstrated *in vitro*, and it has been suggested that erythrocytes may be carriers of the virus. However, the association between HIV particles or viral proteins and erythrocytes in HIV-infected individuals is still to be elucidated.

**Methodology/Principal Findings:**

HIV-positive participants (n = 112) were classified into two groups according to values of three plasma viral loads (pVL) determined during the 12-month period prior to the study. The first group included 71 individuals with detectable pVL, whereas the second group included 41 individuals with undetectable pVL. Plasma viral load, erythrocyte-associated p24-antigen and p24-antigen in plasma were determined at the moment of the study. A total of 51 out of the 71 patients with detectable pVL showed erythrocyte-associated p24-antigen whereas 13 showed p24-antigen in plasma. Twenty-two out of the 51 patients with erythrocyte-associated p24-antigen showed pVL<10,000 copies/ml and undetectable p24-antigen in plasma. The data indicates that the amount of erythrocyte-associated p24-antigen was not related to p24-antigen in plasma or pVL levels in this group. Among the 41 patients with prior undetectable pVL, eight presented detectable pVL and erythrocyte-associated p24-antigen at the moment of the study. The other 33 showed undetectable pVL and five of these presented erythrocyte-associated p24-antigen. A positive relationship was found between the presence of erythrocyte-associated p24-antigen and the detectable pVL at the moment of the study (*p*<0.00001). Even more, in another series of assays, a detectable viral load associated to erythrocytes was determined and it was always accompanied by erythrocyte-associated p24-antigen detection.

**Conclusions/Significance:**

This study demonstrates the presence of erythrocyte-associated p24-antigen in HIV-infected individuals. Since erythrocyte-associated p24-antigen is not always related to pVL or p24-antigen in plasma, erythrocyte-associated p24-antigen showed viral expression not represented in plasma. Therefore, the determination of erythrocyte-associated p24-antigen may contribute to better understand the kinetics and/or evolution of HIV infection.

## Introduction

The concept of immune-adherence to describe the antibody- and complement-mediated binding of bacteria to erythrocytes was introduced by Nelson in 1953 [1]. Immune complexes, namely antigens opsonized by antibodies and complement, are bound to erythrocytes through specific interactions of the complement protein C3b with the erythrocyte complement receptor 1 (CR1 or CD35) in the erythrocyte membrane. Erythrocytes carried in the bloodstream pass through the spleen and liver where macrophages take up the immune complexes [2], [3]. During persistent chronic viral infections, simultaneous antibody and virus production occurs, creating the conditions for the formation of immune complexes and the subsequent erythrocyte adherence. However, erythrocyte-associated antigen is not usually examined in chronic viral infections.

Human immunodeficiency virus type 1 (HIV) particles are known to be capable of binding to erythrocytes *in vitro* by at least three mechanisms: 1) immune complexes may bind to the CR1 receptor by means of complement proteins, 2) HIV may bind to CR1 on the erythrocyte membrane by means of complement proteins in the absence of antibodies, and 3) HIV can bind to Duffy antigen receptor for chemokines (CD55 or DARC) present in erythrocytes. This binding of HIV to erythrocytes *in vitro* needs the presence of Ca^2+^/Mg^2+^ [4]–[7]. It has been observed that HIV-positive individuals show increased C3 activation and decreased number of CR1 on erythrocyte membranes [8], [9]. This is likely evidence of the immune adherence *in vivo*. Erythrocyte-associated HIV may play an important role as an intermediary of infection of T lymphocytes, monocytes, macrophages from spleen and liver and other susceptible cells [6], [7], [10]–[12].

The association between HIV particles or viral proteins and erythrocytes in HIV-infected individuals is still to be elucidated. To date, only two studies on erythrocyte-associated HIV RNA in infected individuals have been published, with controversial conclusions. In the first study, HIV RNA bound to erythrocytes was observed in HIV-positive individuals even in some with undetectable plasma viral load (pVL) during highly active antiretroviral therapy (HAART) [11]; in the second study, erythrocyte-associated HIV RNA was not found in the group of individuals under HAART [13]. These two studies did not take into account the possible presence of free viral antigen regardless of the presence of viral particles. The presence of p24-antigen in HIV infection is a marker of viral expression. Consequently, assessment of viral antigens may provide important information on follow-up and infection evolution.

The aim of this work was to study erythrocyte-associated viral antigen in HIV-positive individuals at different stages of the infection. Here we present evidence indicating that erythrocyte-associated p24-antigen (Ag-E) is present in HIV-positive individuals even in some with undetectable p24-antigen or viral load in plasma. Indeed the presence of Ag-E was not always related to the pVL or p24-antigen in plasma. The study of HIV bound to erythrocytes is important in order to improve our understanding of the kinetics of HIV infection and because of its potential usefulness as a tool for the follow-up of patients.

## Results

### p24-antigen is detected in erythrocytes of HIV-positive individuals

In order to search for p24-antigen in purified erythrocytes from HIV-positive individuals, samples of 71 individuals with detectable pVL (≥50 copies per ml) in at least one sample during the year prior to the study were used. Ag-E was found in 71.8% (51/71) of the individuals, whereas p24-antigen in plasma (Ag-P) was found only in 18.3% (13/71) of them. Examination of possible combinations between Ag-E and Ag-P showed the presence of Ag-E with no Ag-P in 56.3% (40/71) of HIV-positive individuals, absence of both Ag-E and Ag-P in 25.4% (18/71) and presence of both in 15.5% (11/71). Only 2.8% of patients (2/71) showed presence of Ag-P in the absence of Ag-E (**Table 1**). In all cases the median and interquartile range (IQR) were calculated considering only detectable values. A median level of 41 pg p24-antigen/ml was found in purified erythrocytes (IQR 21–121). In plasma, median values of p24-antigen were 148 pg/ml (IQR 105–152).

**Table 1:** Plasma viral load and p24-antigen in plasma and erythrocytes from individuals with detectable plasma viral load in at least one sample during the year prior to the study.

HIV-positive individuals	pVL (copies/ml)	Ag-E (pg/ml)	Ag-P (pg/ml)	HIV-positive individuals	pVL (copies/ml)	Ag-E (pg/ml)	Ag-P (pg/ml)
1	<50	>152	NR	36	13,888	NR	NR
2	<50	>152	NR	37	14,293	NR	NR
3	<50	NR	NR	38	16,334	134.48	NR
4	<50	112.4	NR	39	17,489	24.88	NR
5	53	NR	NR	40	17,781	29.93	NR
6	67	<20*	NR	41	18,188	28.08	NR
7	68	<20*	NR	42	19,522	21	NR
8	76	>152	NR	43	22,061	NR	NR
9	83	<20*	NR	44	22,217	126	>152
10	90.6	97.36	NR	45	25,334	117.6	NR
11	104	NR	NR	46	27,478	NR	148
12	109	NR	NR	47	30,585	33	NR
13	126	34.52	NR	48	31,627	NR	NR
14	131	<20*	NR	49	39,441	<20*	NR
15	131	>152	NR	50	41,687	21.64	40
16	135	NR	NR	51	45,594	97.2	NR
17	144	>152	NR	52	46,347	NR	NR
18	175	<20*	NR	53	48,125	33.16	NR
19	398	<20*	NR	54	50,394	<20*	NR
20	479	30.4	NR	55	51,107	30	NR
21	1,389	<20*	NR	56	62,679	NR	NR
22	1,406	103.2	NR	57	100,928	>152	>152
23	1,603	NR	NR	58	155,010	57	NR
24	1,720	<20*	NR	59	160,981	27.2	90
25	2,048	NR	NR	60	198,000	121.12	>152
26	3,087	NR	NR	61	247,521	61	127.17
27	3,966	<20*	NR	62	261,100	NR	NR
28	4,133	20.72	NR	63	316,443	107.2	NR
29	4,445	60.72	NR	64	380,691	37.76	NR
30	5,207	NR	NR	65	426,691	NR	NR
31	9,031	59	NR	66	>500,000	45.08	126
32	9,329	NR	NR	67	>500,000	NR	120
33	11,422	41	NR	68	>500,000	>152	>152
34	12,855	23.04	NR	69	>500,000	>152	NR
35	13,100	103.6	34.4	70	>500,000	>152	>152
				71	>500,000	>152	>152
					**Median**	**41**	**148**
					**IQR**	**21–121**	**105–152**

**pVL:** plasma viral load at the moment of the study.

**Ag-E (pg/ml):** pg erythrocytes-associated p24-antigen per milliliter of purified erythrocytes.

**Ag-P (pg/ml):** pg p24-antigen per milliliter in plasma.

**NR:** non reactive.

**Median:** median was calculated having in mind the detectable values.

**IQR:** interquartile range. In all cases used, IQR was calculated considering only detectable values.

*: this value was considered positive because it was under 20 pg but over the cut-off (value<20).

In addition, we found pVL<10,000 copies/ml in 32 out of the 71 patients evaluated and pVL≥10,000 copies/ml in the remaining 39. No p24-antigen in plasma was detected in any of the 32 individuals with pVL<10,000 copies/ml, while Ag-E was detected in 22 out of these 32 individuals. Indeed, four out of these 22 individuals presented pVL<50 copies/ml, and three out of these four exhibited Ag-E levels above 110 pg p24-antigen/ml. Eleven out of the 39 individuals with pVL≥10,000 copies/ml presented both Ag-E and Ag-P, whereas two showed absence of Ag-E and presence of Ag-P and 18 showed presence of Ag-E without detectable Ag-P. Finally, p24-antigen was not detected at all in the remaining eight individuals (**Table 1**). To note, eight out of the 13 cases where Ag-P was detectable showed higher levels of Ag-P than of Ag-E (**Table 1**).

It is important to highlight that p24-antigen was never detected in the supernatant of the last wash of purified erythrocytes (see Methods section), showing that Ag-E determination was not affected by Ag-P.

**Figure 1:**
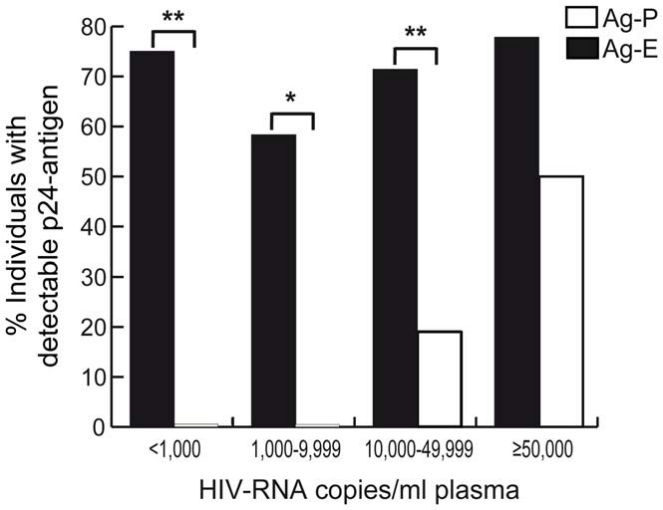
p24-antigen associated to erythrocytes is not related to plasma viral load. Seventy one individuals with detectable plasma viral load (pVL) in at least one sample during the year before the study were classified into four intervals according to their pVL at the moment of the study: 1) <1,000 copies/ml (n = 20), 2) 1,000–9,999 copies/ml (n = 12), 3) 10,000–49,999 copies/ml (n = 21) and 4) ≥50,000 copies/ml (n = 18). Bars represent percentages of subjects with p24-antigen associated to erythrocytes (Ag-E, black bars) and p24-antigen in plasma (Ag-P, white bars). In each of the first three intervals there is a significant difference between the presence of Ag-E and Ag-P. No relationship is observed between Ag-E and pVL among the different pVL intervals (*p* = 0.68). A significant relationship was found between pVL intervals and the presence of Ag-P (*p*<0.0001). * p<0.001; ** p<0.0001.

With the aim to elucidate the relationship between pVL and the presence of Ag-E and Ag-P, the 71 individuals studied were assigned to four groups according to their pVL at the moment of the study: 1) <1,000 copies/ml (n = 20), 2) 1,000–9,999 copies/ml (n = 12), 3) 10,000–49,999 copies/ml (n = 21), 4) ≥50,000 copies/ml (n = 18), and the percentage of individuals with p24-antigen detection in plasma and in erythrocytes was evaluated (**Figure 1**). As expected, there was a significant positive relationship between the different groups of pVL and the presence of Ag-P (*p*<0.0001), where Ag-P was related to higher pVL, being detectable in individuals with pVL≥10,000 copies/ml (*p*<0.0001). To note, Ag-P was not always detected in individuals with pVL≥10,000 copies/ml. On the other hand, a high proportion of individuals (more than 50% in every group) presented Ag-E regardless of their pVL (*p* = 0.68). Moreover, no relationship was observed between the presence of Ag-E and Ag-P as reported above (**Table 1**).

In summary, these results demonstrate the existence of Ag-E in HIV-positive individuals. Moreover, the presence of p24-antigen in erythrocytes was not necessarily related to Ag-P or pVL, even in those patients with pVL<10,000 copies/ml and absence of Ag-P. Ag-E was present in more than 50% of these cases.

### p24-antigen associated to erythrocytes was detected in HIV-positive individuals with undetectable plasma viral load for one year

After demonstrating the presence of p24-antigen in purified erythrocytes from HIV-positive individuals, it was important to note that we found this Ag-E in patients with pVL<1,000 copies/ml at the moment of the study. Therefore, we wondered whether Ag-E might also be detected in individuals with undetectable pVL for one year prior to the study. To this end, we used 41 samples from HIV-positive individuals with undetectable pVL in three determinations for 12 months before the study. Eight out of the 41 individuals showed detectable pVL (median: 112 copies/ml; IQR 77–403) at the moment of the study and all of them presented Ag-E (median: 25 pg p24-antigen/ml; IQR 20–76) (**Table 2**). In contrast, 33 out of the 41 individuals presented undetectable pVL at the moment of the study, and five out of these 33 showed Ag-E (median: 38 pg p24-antigen/ml; IQR 31–97). When analyzing both groups, a significant positive relationship between detectable pVL and the presence of Ag-E was found (*p*<0.00001) (**Table 2**). p24-antigen was not detected in plasma of any sample or in the supernatant of the last wash of each sample of purified erythrocytes from the 41 individuals.

**Table 2:** p24-antigen associated to erythrocytes in individuals with consecutive undetectable plasma viral load for one year.

pVL	HIV-positive individuals	pVL (copies/ml)	Ag-E (pg/ml)	Ag-P (pg/ml)
**Detectable**	1	67	<20*	NR
**N = 8**	2	76	>152	NR
	3	83	<20*	NR
	4	98	90.6	NR
	5	126	34.52	NR
	6	175	<20*	NR
	7	479	30.4	NR
	8	3,966	<20*	NR
**Undetectable**	9 to 36	<50	NR	NR
**N = 33**	37	<50	30.4	NR
	38	<50	31.6	NR
	39	<50	38.0	NR
	40	<50	42.0	NR
	41	<50	>152	NR

**pVL:** plasma viral load at the moment of the study.

**Undetectable pVL:** plasma viral load <50 copies per milliliter.

**Detectable pVL:** plasma viral load ≥50 copies per milliliter.

**Ag-E (pg/ml):** pg erythrocytes-associated p24-antigen per milliliter of purified erythrocyte.

**Ag-P (pg/ml):** pg p24-antigen per milliliter in plasma.

**NR:** non reactive.

*: this value was considered positive because it was under 20 pg but over the cut-off (value<20).

These results show that Ag-E was present in individuals with undetectable pVL for one year. Furthermore, pVL detection at the moment of the study is related to the presence of Ag-E, suggesting that Ag-E determination could be a possible indicator for viral expression.

### Erythrocyte-associated p24-antigen quantification is independent of the erythrocyte purification method used

With the aim to determine whether the quantification of Ag-E depends on the purification method used, erythrocyte-enriched fractions from 27 HIV-positive individuals were divided into two aliquots. One aliquot was purified by dextran sedimentation while the other was purified by magnetic bead depletion (magnetic beads coated with antibodies to CD4 and CD8). By dextran sedimentation, samples were depleted of leukocytes in 93.6% as compared to whole blood (median residual leukocytes: 175/µl; median residual lymphocytes: 58/µl; median residual CD3+ CD4+ lymphocytes: 27/µl). On the other hand, the leukocyte depletion by magnetic beads was 98.0% as compared to whole blood (median residual leukocytes: 100/µl; median residual lymphocytes: 11/µl; median residual CD3+ CD4+ lymphocytes: 3/µl). The maximum Ag-E difference between both procedures was 21.0%, with a mean value of 5.0%. Linear regression between the Ag-E levels obtained by dextran sedimentation and those obtained by magnetic bead depletion showed an R^2^ of 0.981 and a slope of 0.972. Ag-E was detectable by both techniques in 17 samples, whereas it was undetectable by both techniques in other 10 samples. Besides, in erythrocyte-enriched fractions of six samples from HIV-positive individuals purified by magnetic bead depletion using anti-CD3 antibody coated beads, a leukocyte depletion of 99% was observed. Three out of these six samples showed detectable pVL and an Ag-E higher than 152 pg/ml, while the other three had neither pVL nor Ag-E. Therefore, these experiments suggest that determination of p24-antigen on erythrocytes yields comparable results regardless of the purification method used.

### Contaminating leukocytes are not significant sources of p24-antigen in purified erythrocytes

In order to elucidate whether residual leukocytes from purified erythrocytes were significant sources of p24-antigen, leukocytes (Leukocytes _HIV+_) and erythrocytes (E _HIV+_) from seven HIV-positive individuals with detectable Ag-E were obtained by dextran sedimentation. The residual leukocytes of each sample were quantified in purified erythrocytes. Then, the same amount of quantified contaminant leukocytes was mixed with erythrocytes from HIV-negative individuals (Leukocytes _HIV+_ + E _HIV−_). Subsequently, both samples from each individual, (E _HIV+_) and (Leukocytes _HIV+_ + E _HIV−_), were subjected to p24-antigen determination as described in the Methods section. No p24-antigen was detected in any of the Leukocytes _HIV+_ + E _HIV−_ samples, in remarkable contrast with the E _HIV+_ samples, which were all positive for p24-antigen detection (data not shown). All data presented here indicate that if there is p24-antigen associated with residual leukocytes, it is not a significant contributor to Ag-E determination.

### A defined population of erythrocytes is responsible for carrying the p24-antigen associated to their membranes

**Figure 2:**
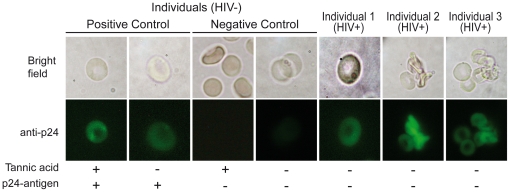
p24-antigen is associated to erythrocyte membrane in HIV-positive individuals. Purified erythrocytes from HIV-negative and HIV-positive individuals were subjected to immunofluorescence with anti-p24 antibody. Homogeneous fluorescence in membrane is observed in purified erythrocytes from HIV-positive individuals (the three images shown are representative of 12 different HIV-positive individuals with plasma viral loads ranging from 1,000 to 20,000 copies/ml). Purified erythrocytes from HIV-negative individuals incubated with p24-antigen alone or p24-antigen plus tannic acid were used as positive control. No positive fluorescence is observed in purified erythrocytes from HIV-negative individuals.

To further confirm that p24-antigen was really associated to the erythrocyte membrane, we performed a p24-antigen immunofluorescence in purified erythrocytes from 15 HIV-positive individuals with pVL ranging from 1,000 to 20,000 copies/ml. Twelve out of the 15 individuals presented a p24-antigen positive signal on erythrocytes with a very similar pattern. **Figure 2** shows positive p24-antigen immunoreactivity on erythrocytes from three of the 12 positive patients. Erythrocytes from HIV-negative individuals treated or not with tannic acid (positive controls) were able to bind recombinant p24-antigen. In contrast, no fluorescence was detected in purified erythrocytes from HIV-negative individuals used as negative controls (**Figure 2**).

**Figure 3:**
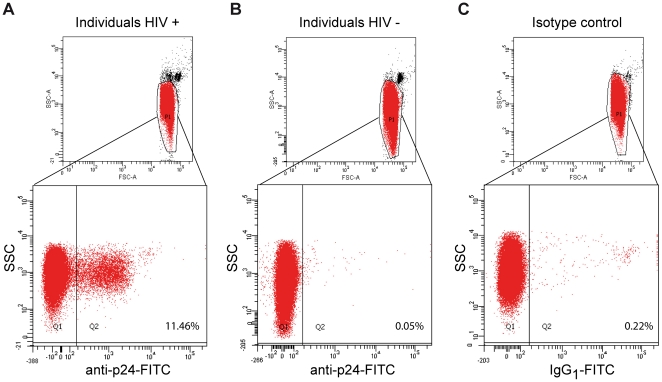
p24-antigen is associated to a defined erythrocyte population. Representative flow-cytometry analysis of p24-antigen immunostaining in purified erythrocytes is shown and side scatter (SSC) vs. FITC fluorescence is plotted. (A) Purified erythrocytes from HIV-positive individuals and stained with anti-p24-FITC exhibited a mean value of 14±4% p24-antigen-positive erythrocytes (mean fluorescence of 33±5). (B) Representative purified erythrocytes from HIV-negative individuals were stained with the anti-p24-FITC, and no specific stain in erythrocytes was found. (C) Isotype control for HIV-positive purified erythrocytes. Inserts show the corresponding dot plot and region P1 represents FSC/SSC light-scatter gate. Percentage of positive events is shown in the Q2 quadrant. Graphs are representative of seven independent experiments (for HIV-positive experiments samples from individuals with plasma viral loads between 1,000 and 20,000 copies/ml were used).

With the purpose of determining the proportion of erythrocytes carrying p24-antigen, we performed flow-cytometry assays using a FITC-conjugated p24-specific antibody. Erythrocytes of HIV-positive individuals with pVL between 1,000 and 20,000 copies/ml showed a mean of p24-antigen-immunoreactive erythrocytes of 14±4% (mean ± SD) with a mean fluorescence of 33±5 (mean ± SD) (**Figure 3A**). It is interesting to note that p24-antigen was associated to a defined erythrocyte population. Only background or unspecific fluorescence was detected in the overall erythrocyte population from HIV-negative individuals (**Figure 3B**).

In conclusion, these results demonstrate that p24-antigen is associated to the membrane of erythrocytes within a defined population of erythrocytes in HIV-positive individuals.

### Viral load associated to erythrocytes is detected in HIV-positive individuals with p24-antigen associated to erythrocytes

The results shown so far reveal the presence of Ag-E in patients with different pVL. This finding led us to wonder whether the detection of p24-antigen on erythrocytes is related to viral HIV RNA in these cells. To clarify this issue viral load associated to erythrocytes (eVL), pVL, and Ag-P were determined in 34 HIV-positive individuals (10 with undetectable Ag-E and 24 with detectable Ag-E). **Table 3** shows that 8 out of the 10 patients with undetectable Ag-E had no viral HIV RNA in plasma, or on erythrocytes and no Ag-P. There were two patients with detectable pVL but undetectable Ag-E, Ag-P and eVL. Results corresponding to the individuals with detectable Ag-E showed that 7 out of the 24 patients showed only Ag-E, being the only test that reflected viral expression. Interestingly, three out of the Ag-E positive individuals (patients 18, 19 and 20) were accompanied only by the presence of eVL, showing that viral expression is reflected only in erythrocytes (**Table 3**). Five individuals presented only Ag-E and presence of pVL. Finally in nine of the patients with detectable Ag-E, HIV RNA was found in both plasma and erythrocyte fractions. Three out of these nine patients were the only ones with detectable Ag-P, corresponding with the highest pVL.

**Table 3:** Viral load associated to erythrocytes in HIV-positive individuals.

Ag-E	HIV-positive individuals	pVL (copies/ml)	Ag-P (pg/ml)	eVL (copies/ml)	Ag-E (pg/ml)
**Undetectable**	1 to 8	<50	NR	<50	NR
**N = 10**	9	2,060	NR	<50	NR
	10	6,624	NR	<50	NR
**Detectable**	11	<50	NR	<50	<20*
**N = 24**	12	<50	NR	<50	<20*
	13	<50	NR	<50	<20*
	14	<50	NR	<50	<20*
	15	<50	NR	<50	80
	16	<50	NR	<50	80
	17	<50	NR	<50	92
	18	<50	NR	74	28
	19	<50	NR	128	<20*
	20	<50	NR	368	<20*
	21	150	NR	<50	<20*
	22	279	NR	<50	29
	23	1,039	NR	<50	<20*
	24	1,131	NR	<50	<20*
	25	2,417	NR	<50	108
	26	111	NR	215	96
	27	1,326	NR	104	<20*
	28	2,672	NR	830	<20*
	29	11,581	NR	71	80
	30	26,355	NR	64	104
	31	45,032	NR	1,268	<20*
	32	52,340	96	235	80
	33	>500,000	>152	3,823	<20*
	34	>500,000	>152	14,364	33

**pVL:** plasma viral load at the moment of the study.

**Undetectable Ag-E:** undetectable erythrocytes-associated p24-antigen.

**Detectable Ag-E:** detectable erythrocytes-associated p24-antigen.

**Ag-E (pg/ml):** pg erythrocytes-associated p24-antigen per milliliter of purified erythrocyte.

**Ag-P (pg/ml):** pg p24-antigen per milliliter in plasma.

**NR:** non reactive.

*: this value was considered positive because it was under 20 pg but over the cut-off (value<20).

Although it is unusual to find detectable p24-antigen in plasma samples from patients with undetectable or very low pVL in the erythrocyte fraction, Ag-E detection was possible with low amounts of eVL. These results suggest that the presence of eVL could be related to a detectable Ag-E.

## Discussion

The presence of viral proteins in HIV infection is an evidence of viral expression regardless of the presence of viral particles. There are other sources of p24-antigen including degraded virions or defective particles and those released by killed infected cells [14]. Consequently, assessment of viral antigens may provide important information on infection evolution. In this direction, erythrocytes could provide a good target to assay the presence of viral antigens. Moreover, research on antigen and/or virus carried by erythrocytes may be relevant during HIV infection due to the large number of circulating erythrocytes that would facilitate infection of susceptible cells [6], [7], [10]–[12]. The present study reports the presence, sometimes in high values, of p24-antigen associated to erythrocytes in HIV-positive individuals, even in those with undetectable pVL.

Using dextran sedimentation, Hess *et al.*, 2002 [11] reported detectable viral loads in purified erythrocytes in a group of patients under HAART, while, in a similar work but using beads-mediated CD3 depletion as an erythrocyte purification technique, Fierer *et al.*, 2007 [13] did not reach the same conclusion and suggested that the results obtained by Hess and colleagues might arise from a CD3+CD4+ lymphocyte contamination. However, in Fierer's study, the presence of HIV-RNA in CD3+ cells was not evaluated.

Here we present evidence that partly elucidates the controversy about HIV association to erythrocytes in HIV-positive individuals. Although the viral RNA detection assay is quite sensitive, quantification of the p24 viral antigen allowed us to measure not only the antigen from viral particles but also the free p24-antigen.

In order to determine whether p24-antigen is present in erythrocytes of HIV-positive individuals, it was necessary to rule out the presence of p24-antigen associated to other non-erythrocyte cells or from traces of plasma in the purified erythrocyte fraction. To rule out potential confounds arising from the use of different purification methods [11], [13], we compared the efficiency of erythrocyte purification by dextran sedimentation and by magnetic bead depletion. Our results showed that p24-antigen quantification on erythrocytes was similar with the two methodologies. We included the use of CD8+ beads because Gulzar *et al.*, 2008 [15] reported a significantly higher proportion of cells with expression of CD8+ HIV-1 (gag)+ rather than of CD4+ HIV-1 (gag)+ T-cells. Besides, we also tested purification by magnetic bead depletion with antibodies to CD3 and detected high levels of Ag-E. Even more, the same amount of residual leukocytes in purified erythrocytes from HIV-positive individuals was not capable of achieving that the p24-antigen detection in purified erythrocytes from HIV-negative individuals be over the assay's cut-off. Therefore, we may state that measurement of p24-antigen on purified erythrocytes is not due to contamination and is not affected by the purification method chosen.

Horakova *et al.*, 2004 [4] and Beck *et al.*, 2009 [7] described EDTA-mediated *in vitro* inhibition of HIV binding to erythrocytes in the presence and absence of complement respectively. We confirmed these results in our laboratory (data not shown). The possible binding of antigen present in plasma to erythrocytes after blood sample collection was prevented by the use of EDTA as anti-coagulant. Moreover, since p24-antigen was not detected in the last erythrocyte-purification wash, we discarded the possibility that Ag-E originates from contamination of other cell types or from plasma.

Among the 71 individuals with detectable pVL in at least one sample during the year preceding the present study, a high percentage (71.8%) showed Ag-E and the amounts of Ag-E varied widely among individuals with similar pVL values. Ag-E levels were not related to pVL values or to the presence and/or levels of Ag-P. This can be explained by the possibility that erythrocytes of each individual may have different virus adsorption capacities. This is supported by a recent work where virus binds *in vitro* to erythrocytes of HIV-negative individuals in a wide range of absorption capacities [7]. Another possible explanation would be given by the several factors that may vary in each HIV-positive individual, for example, the presence of antibodies which can mask the viral antigen, a different activity of complement factors as shown by Taush *et al.*, 1986 [8] (increased C3 activation in HIV-positive individuals), different kinetics of viral clearance and/or the amount of receptors available on the erythrocyte membrane [16].

Individuals with pVL<10,000 copies/ml (**Table 1**) showed only Ag-E detectable and Ag-P undetectable, suggesting that the erythrocytes of these individuals have the capacity of adsorbing a great deal of the antigen produced. In contrast, individuals presenting pVL≥10,000 copies/ml presented Ag-P even in higher quantities than Ag-E, suggesting that in these individuals viral production may exceed adsorption capacity of erythrocytes, allowing the virus to reach high values in plasma. This was the case of the two individuals with undetectable Ag-E but with high levels of p24-antigen in plasma. However, 18 individuals of this group showed Ag-E and absence of Ag-P.

As regards the eight individuals with previous undetectable pVL for 12 months who presented current detectable pVL at the moment of the study, all of them also showed a positive detection of erythrocyte-associated p24 antigen. As shown in **Table 2**, the pVL in these individuals were relatively low, suggesting a possible recent viral rebound, while the values of Ag-E were similar to those found in patients with previous detectable pVL (**Table 1**). A highly positive significant relationship was found between a first detectable pVL after 12 months of undetectable pVL and the presence of Ag-E. Our results suggest that the presence of Ag-E could be caused by a rebound marker in pVL, suggesting that pVL might be assessed during the follow-up of these patients only when the antigen is present on erythrocytes, thus reducing monitoring costs.

Eight of the individuals shown in Tables 1 and 2 (71 and 41 respectively) showed detectable Ag-E and undetectable pVL at the moment of the study. In four of these, the Ag-E value was >110 pg p24-antigen/ml. Considering that the circulating erythrocytes have a mean life of 120 days, two or three rounds of erythrocyte renewal should have taken place in them since the last plasma RNA detection. Since p24-antigen and HIV RNA were absent in the plasma of these individuals, the origin of Ag-E is unknown. A possible explanation to this may be addressed by the results of Schüpbach *et al.*, 2005 [17], who found a discrepancy between the values of plasma viral antigen mass and pVL. Moreover, most p24-antigen present in plasma from infected patients cannot be pelleted [18], demonstrating that it is located outside virus particles. This result has been confirmed in the samples examined in our laboratory [19]. Schüpbach *et al.*, 2005 [17] postulated that p24-antigen may be derived from virus replication in reservoirs not represented by plasma HIV RNA. If that assumed by Schüpbach *et al.*, 2005 [17] and our results are correct, it could be expected that Ag-E will be found in individuals who show undetectable HIV RNA in plasma. Therefore, most of the p24-antigen found in erythrocytes would be originated in viral reservoirs, bound to erythrocytes, and remaining undetected in plasma. Indeed, Ag-E could come from latently infected cells, which, when stimulated, release virus to plasma, which is likely adsorbed by erythrocytes [20]. Alternatively, erythrocytes in close contact with liver and spleen macrophages could manage to adsorb antigens from them. In addition, Prado *et al.*, 2004 [21] suggested that p24-antigen binding to erythrocytes in HIV-positive individuals during treatment interruptions may explain the lack of longitudinal intrapatient correlation between Ag-P and pVL levels. Erythrocyte-associated p24-antigen would reflect continuous viral replication even in patients with undetectable pVL.

In support of our findings, we also demonstrated the association between p24-antigen and erythrocytes by immunofluorescence and flow-cytometry in patients with lower or moderate pVL levels. Our data indicate that Ag-E does not seem to be present in all erythrocytes, since only 14±4% erythrocytes showed positive p24-antigen in flow cytometry assays (**Figure 3**). This last observation may be partially explained by incomplete viral clearance in erythrocytes recently associated to p24-antigen, or by heterogeneity in the number of receptors available.

Our studies reveal that the presence of Ag-E is not necessarily associated with detectable eVL. However, the presence of eVL was always accompanied by Ag-E detection. This fact, together with the low eVL and pVL observed in some patients with detectable Ag-E, suggests that p24-antigen detected in the erythrocyte fraction is mostly not associated with viral particles. This could be explained by the fact that the transfer of virus to other cells and/or efficient viral clearance may contribute to diminishing eVL [11], [12]. Besides, eVL is detected in most of the patients who have detectable pVL, as well as in patients with undetectable pVL (**Table 3**).

The results presented in this study demonstrate the presence of Ag-E in HIV-positive individuals by three different methods of detection (ELISA, immunofluorescence microscopy and flow cytometry) and that Ag-E is not always related to plasma viral load or to p24-antigen in plasma. In addition, they show the presence of an important amount of viral antigen associated to erythrocytes in individuals undergoing different stages of infection, even in those with undetectable plasma viral load. Finally, the erythrocyte-associated antigen study has a significant potential as a tool for elucidating HIV infection kinetics, evolution, and follow-up of patients.

## Materials and Methods

### Study population

A total of 234 HIV-positive individuals older than 21 years of age who attended the Argentinean National Reference Centre for AIDS for pVL assessment were invited to participate in this study. Sixty-two HIV-negative individuals were recruited as controls. The protocol was approved by the local Ethics Committee of the University of Buenos Aires and a written informed consent was signed by each participant.

A total of 112 HIV-positive participants were classified into two groups according to the values of pVL obtained in three determinations during the 12-month period prior to the current study. The first group included 71 individuals with pVL≥50 copies/ml in at least one sample during that period. The second group included 41 individuals with pVL<50 copies/ml in all the samples obtained during the same period. Samples from other 34 HIV-positive individuals were used to determine the viral load associated to erythrocytes (eVL), whereas samples from the remaining 88 HIV-positive individuals were used as controls and for other assays.

### Sample processing

Samples were obtained by venous puncture using EDTA as anti-coagulant. Erythrocytes were processed the day of blood collection. Blood was centrifuged at 1,400 g for 10 minutes. Plasma was stored for pVL and Ag-P determination. The buffy coat was discarded. A volume of 1 ml of the erythrocyte-enriched fraction was diluted 1/2 in PBS, followed by addition of Dextran 4% (MW 200,000–275,000) (Biochemical), mixed by inversion and kept in vertical position for 30 minutes at RT. The erythrocyte sediment was washed four times in PBS. In each wash, the supernatant and interface layers were aspirated and discarded. As control for the washing process, the supernatant of the last erythrocyte wash (Ag-W) was stored to be used in p24-antigen detection assays. After sedimentation, all procedures were carried out at 4°C. The low temperature conditions allowed for a p24-antigen recovery approximately 6-fold higher than that obtained at RT (data not shown). Median final concentration of erythrocytes in the purified fraction was 7.78 10^6^ erythrocytes/µl (IQR 7.24 10^6^– 8.90 10^6^). A ratio of one leukocyte per 44,000 erythrocytes was estimated.

Alternatively, erythrocyte-enriched fractions from 27 HIV-positive individuals were purified using a mix of magnetic beads coated with antibodies to CD4 and CD8 (BD Biosciences). The same process was repeated using magnetic beads coated with antibodies to CD3 (DYNAL) for 6 samples.

### Erythrocyte-associated viral antigen release

Erythrocytes were subjected to acid treatment in order to release erythrocyte-associated antigen. Then, 750µl glycine-HCl buffer (pH 3.2) was added to 250µl of purified erythrocytes at RT and centrifuged at 470g for 5 minutes. The supernatant was neutralized to pH 7.2 with NaOH 0.1N and kept at −20°C. The glycine-HCl treatment was also performed in plasma and in the supernatant of the last wash (Ag-W) of each sample as a control of erythrocyte washing. p24-antigen was assessed in all fractions.

In order to determine whether the acid treatment affects p24-antigen detection assays, purified erythrocytes from HIV-negative individuals were incubated with a known amount of recombinant p24-antigen and subjected to the glycine-HCl treatment under the same conditions. The acid treatment did not affect p24-antigen measurements (data not shown).

### p24-antigen determination

p24-antigen was quantified by ELISA (Murex HIV antigen Mab, Abbott). Cut-off calculation: a) Ag-P: according to the manufacturer; b) Ag-E: average absorbance obtained after glycine-HCl treatment of purified erythrocytes from 15 HIV-negative individuals plus two Standard Deviations (SD); c) Ag-W: average absorbance of supernatants obtained from Ag-W glycine-HCl buffer treatment of 15 HIV-negative individuals plus two SD. The detection limit for ELISA assay was 5 pg p24-antigen. For quantification, the minimum required was 5 pg p24-antigen in plasma and 20 pg for Ag-E.

### Viral load determination

Plasma viral load was assessed by branched DNA (b-DNA) technology, with a detection limit of 50 HIV-1 RNA copies/ml (Versant HIV-1 RNA 3.0, Siemens Healthcare Diagnostics Inc., Tarrytown, NY). For measurement of erythrocyte-associated HIV load, RNA extraction from one milliliter of purified erythrocytes by magnetic bead depletion was performed using TRIZOL (TRIzol® Reagent, Invitrogen) and subjected to the HIV-1 RNA 3.0 assay, in the RNA capture step. In order to determine whether TRIZOL-mediated RNA extraction affects determination of viral load, purified erythrocytes from HIV-negative individuals were incubated with a known amount of two different viral strains (BaL HIV-1 and HXB2 HIV-1), and subjected to viral load determination. This process did not affect viral load measurements (data not shown).

### Immunofluorescence

Purified erythrocytes were fixed with 4% p-formaldehyde in PBS. Mouse anti-HIV-1 p24 Gag monoclonal antibody (NIH AIDS Research & Reference Reagent Program) and goat anti-mouse IgG-FITC (BD Biosciences) were used as primary and secondary antibodies respectively. Samples were observed in a Nikon Eclipse E200 (Plan100×, N.A. 1.35) epifluorescence microscope. Purified erythrocytes from HIV-negative individuals were incubated with tannic acid to allow antigen binding, then incubated with recombinant p24-antigen (7,500 molecules of p24-antigen/erythrocyte), and used as controls. The recombinant p24-antigen used was the standard positive control of the Murex HIV antigen Mab kit (Abbott). Fifteen different HIV-positive individuals with plasma viral loads between 1,000 and 20,000 copies/ml were used.

### Flow-cytometry

In order to identify scatter properties of erythrocyte population and to ensure the selection of the population of erythrocytes, we stained 10^6^ erythrocytes with glycophorin A, a major transmembrane sialoglycoprotein expressed on human erythrocyte membrane. Afterwards, the erythrocytes were acquired in a flow cytometer and used glycophorin A expression as a marker to define forward and scatter detectors. Acquisition was set by forward/side scatter detectors to log mode which provides better resolution. Once defined forward and scatter detectors, 10^6^ purified erythrocytes from HIV-positive (n = 7) or HIV-negative individuals were stained with monoclonal anti-p24 (FITC-KC57, Coulter Clone, Hialeah, FL) or control isotype antibody. 10^6^ erythrocytes were stained with five microlitres of pre-titrated FITC-KC57. The gate (P1) was defined in the analysis to exclude all other cells and debris, based on forward/scatter dot blot. Analysis was performed using a FACS flow cytometer (FACSCanto, BD) and CellQuest software (BD Biosciences, San Jose, CA).

 CD3+ CD4+ cells in the purified erythrocytes fraction were counted by flow-cytometry (Coulter XL, Coulter Co., Hialeah, FL).

### Statistical analysis

Fisher's Exact Test was used to calculate significance in contingency tables, and the *Chi*-square test for trend was used to evaluate tendencies. A *p*<0.05 was considered significant.

In all cases, the median and interquartile range (IQR) were calculated considering only detectable values.
